# Recent Progress in Structures and Functions of Hepatitis C Virus NS3/4A Proteins

**DOI:** 10.3390/v18020233

**Published:** 2026-02-12

**Authors:** Keyang Huang, Manfeng Zhang, Yihua Huang, Zhongzhou Chen

**Affiliations:** 1 State Key Laboratory of Animal Biotech Breeding, College of Biological Sciences, China Agricultural University, Beijing 100193, China; sz20243020423@cau.edu.cn; 2 State Key Laboratory of Biomacromolecules, Institute of Biophysics, Chinese Academy of Sciences, Beijing 100101, China; 3 University of Chinese Academy of Sciences, Beijing 100101, China

**Keywords:** NS3/4A protease, helicase, unwinding, interdomain allosteric regulation, viral replication, protein engineering, drug resistance

## Abstract

Hepatitis C virus (HCV) chronically infects over 50 million people worldwide and poses a significant risk to global health. The HCV NS3/4A complex, a bifunctional enzyme comprising a protease and a helicase domain, is indispensable for viral replication and immune evasion, making it a pivotal target for direct-acting antiviral agents (DAAs). Here, we summarize its structural features, functional mechanisms, and implications in drug design and protein engineering (e.g., nanopore sequencing applications). The NS3 protease domain is activated by the NS4A cofactor, which mediates viral polyprotein processing and relies on a zinc-binding site for structural stability. The C-terminal helicase domain catalyzes ATP-dependent 3′→5′ unwinding, and allosteric crosstalk between the protease and helicase domains dynamically modulates the enzymatic activity, balancing unwinding velocity and processivity. Beyond supporting viral replication, NS3/4A cleaves MAVS to abolish RIG-I/MDA5 signaling but spares TRIF, leaving TLR3-mediated immunity intact; it also modulates host lipid and iron metabolism, contributing to HCV pathogenesis. Notably, structural and functional studies of NS3/4A lay a solid theoretical foundation for developing novel therapeutic strategies. Currently, DAAs targeting NS3/4A have achieved high sustained virologic response rates; however, resistance-associated substitutions remain a major clinical challenge, particularly in genotype 3 infections. Emerging therapeutic strategies targeting NS3/4A include allosteric inhibition and proteolysis-targeting chimeras (PROTACs)-mediated degradation.

## 1. Introduction

**Figure 1:**
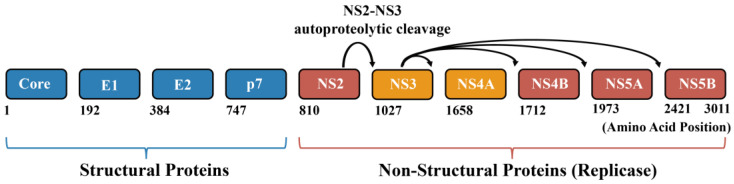
Schematic representation of the Hepatitis C virus (HCV) polyprotein, highlighting the NS3–NS4A protease complex [15]. The HCV genome encodes a single ~3010 amino acid polyprotein that is co- and post-translationally processed into structural proteins (Core, E1, E2, p7) and non-structural proteins (NS2–NS5B). Structural proteins (blue) form the viral particle, while non-structural proteins (red and orange) assemble the replication machinery. The NS3–NS4A complex (orange) is highlighted, illustrating its central role in polyprotein processing, viral replication, and innate immune modulation. Amino acid numbering is indicated below.

Hepatitis C virus (HCV) chronically infects over 50 million individuals worldwide, posing a significant global health risk [1]. Most acute infections (75–85%) progress to chronicity, while only 15–25% resolve spontaneously [2]. Chronic HCV infection drives liver cirrhosis and hepatocellular carcinoma through persistent inflammation and fibrosis [1,3,4,5,6], and also induces disorders of lipid and iron metabolism [7]. Notably, in 2022, viral hepatitis and tuberculosis ranked as the second leading cause of death among communicable diseases, following coronavirus disease 2019 (COVID-19) [8]. HCV exhibits high genetic diversity, with eight major genotypes and numerous subtypes identified to date. Belonging to the *Hepacivirus* genus within the *Flaviviridae* family, HCV has a ~9.6 kb positive-sense single-stranded RNA genome that encodes a ~3011-amino-acid polyprotein precursor. This precursor must undergo proteolytic processing by host signalases and viral proteases (NS2 and NS3/4A) to generate ten mature proteins [9] (Figure 1), including four structural proteins (core, E1, E2, p7) and six non-structural proteins (NS2, NS3, NS4A, NS4B, NS5A, and NS5B).

Among these proteins, the NS3/4A complex has emerged as a cornerstone in both HCV replication and pathogenesis, warranting particular attention in antiviral research. The HCV NS3/4A protease complex, composed of the NS3 protease and its cofactor NS4A, plays a central and multifunctional role in the viral life cycle and pathogenesis, making it a critical focus of research. As a serine protease, NS3/4A cleaves the viral polyprotein to release all mature non-structural proteins (e.g., NS4B, NS5A, and NS5B), a step essential for initiating viral replication. Furthermore, the C-terminal region of the NS3 harbors the NTPase/helicase activity, which is crucial for viral RNA genome replication. Critically, NS3/4A cleaves MAVS, abolishing RIG-I and MDA5 signaling, while leaving the TLR3-TRIF pathway intact, thereby suppressing interferon production [10]. These multifaceted functions make NS3/4A a prime target for the development of direct-acting antiviral agents (DAAs) against HCV [11,12,13,14,15].

In this review, we focus on recent structural and mechanistic insights into the HCV NS3/4A complex, highlighting how its architecture underpins enzymatic function, immune evasion, and therapeutic targeting, with an emphasis on implications for antiviral drug design and resistance.

## 2. Functions of NS3/4A Complex

### 2.1. Viral Replication

NS3/4A forms a central catalytic core of the HCV replication complex, linking polyprotein processing to the initiation of viral RNA replication. This heterodimeric enzyme exhibits multiple coordinated activities, including serine protease, RNA helicase, and NTPase functions [16,17]. The NS3 protease, activated by NS4A, cleaves four polyprotein junctions (NS3/NS4A, NS4A/NS4B, NS4B/NS5A, and NS5A/NS5B) [4,18,19]. These cleavages release mature non-structural proteins essential for membrane-associated replication factories. Precise temporal and spatial control of processing ensures proper replicase assembly, directly coupling proteolysis to replication competence [20,21].

After replicase assembly, the C-terminal helicase/NTPase domain unwinds double-stranded viral RNA in a 3′→5′ direction. It couples ATP hydrolysis to strand separation, driving processive genome replication [4,22]. Single-molecule analyses reveal that NS3 engages RNA through discrete ATP-dependent steps, functioning as a molecular motor [23]. By coordinating proteolytic maturation with RNA remodeling, NS3/4A orchestrates both initiation and maintenance of HCV RNA synthesis.

### 2.2. Immune Evasion

Beyond viral polyprotein processing, NS3/4A also extensively intervenes in cellular processes by cleaving key host proteins. It efficiently degrades MAVS, completely suppressing RIG-I/MDA5-mediated cytoplasmic RNA sensing. In contrast, NS3/4A does not effectively cleave TRIF in hepatocytes, leaving TLR3 signaling functional and contributing to the residual innate immune response observed during HCV infection. This selective disruption of cytoplasmic but not endosomal pathways creates an immunological balance that may facilitate viral persistence while avoiding complete immune silencing [10].

### 2.3. Metabolic Modulation

In lipid metabolism, NS3/4A enhances phosphatidylcholine synthesis in a protease activity–dependent but indirect manner by relocalizing CTP-phosphocholine-cytidyl transferase α (CCTα) from the nucleus to viral replication compartments, thereby locally activating the Kennedy pathway and remodeling membrane lipid composition and fluidity [24]. Concurrently, NS3/4A cleaves the lipid droplet–associated factor Spartin (SPG20), leading to impaired ubiquitination and degradation of adipose differentiation-related protein (ADRP). The resulting stabilization of ADRP drives lipid droplet expansion, thereby promoting a lipid environment that supports HCV replication [25]. Furthermore, NS3/4A cleaves the iron exporter ferroportin 1 (FPN1), leading to intracellular iron accumulation. This event not only exacerbates oxidative stress and the risk of liver injury but also elevates the NTP/dNTP ratio, which benefits viral replication [7,26].

### 2.4. Interdomain Allosteric Regulation

The catalytic activity of full-length NS3/4A differs from that of the single helicase domain, providing evidence for functional interdependence between these two domains. As summarized in Table 1, comparisons of full-length NS3/4A, isolated domains, and interface mutants reveal substrate-dependent, bidirectional coupling between the protease and helicase domains. Interface disruption increases protease activity and DNA unwinding but reduces helicase processivity. In contrast, RNA unwinding is largely interface-independent yet requires the protease domain. Together, these effects support dynamic interdomain communication within NS3/4A (Table 1) [27].

Disrupting the interdomain interface significantly enhances DNA-unwinding rates. Interface mutants (e.g., triple helicase mutant S1483A-M1485A-V1524A) exhibit up to 11.6-fold higher activity than the wild-type full-length protein. Single NS3 helicase also unwinds faster than the full-length NS3/4A (up to 5.7-fold). However, this gain in velocity is offset by reduced processivity under single-turnover conditions, indicating that the protease domain enhances helicase processivity [27].

For RNA unwinding, the full-length protein exhibits significantly higher activity than the single helicase domain. Specifically, the NS3 helicase domain unwinds RNA approximately 10-fold more slowly than the full-length NS3/4A. Interface mutations have negligible effects on RNA unwinding. This observation suggests that the protease domain acts as a functional cofactor for the helicase. Specifically, it stabilizes protein–RNA interactions and modulates unwinding activity through mechanisms that are independent of the interdomain structural interface [27,28].

## 3. Structures of NS3/4A Complex

### 3.1. Protease Domain

**Figure 2:**
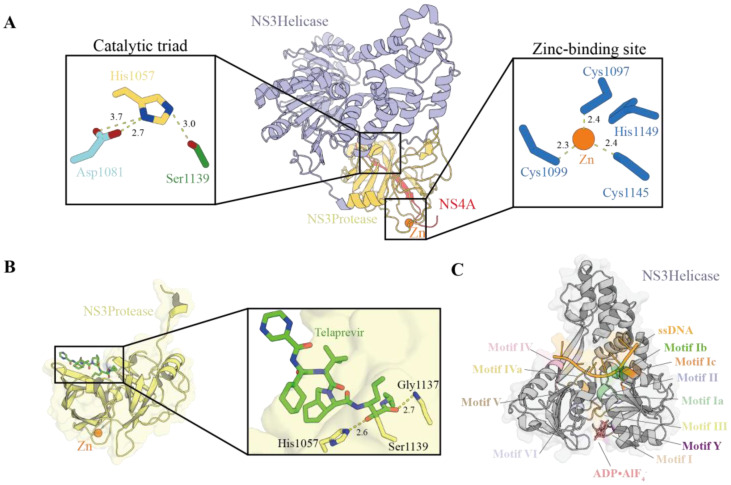
Structural architecture of the HCV NS3/4A complex and its catalytic mechanisms [9,35,36]. (**A**) Crystal structure of the full-length HCV NS3/4A complex (PDB ID: 1CU1) depicting the N-terminal serine protease domain (yellow) and the C-terminal RNA helicase domain (purple). The NS4A cofactor peptide (red) forms a β-strand that integrates into the protease domain, providing essential structural and catalytic support. Inset panels highlight the catalytic triad (left; His1057, Asp1081, and Ser1139) positioned within the substrate-binding cleft, and the zinc-binding site (right) comprising four coordinating residues (Cys1097, Cys1099, Cys1145, and His1149) that stabilize the protease structure. Distances shown are in angstroms. (**B**) Detailed view of the NS3 protease domain in complex with the inhibitor telaprevir (PDB ID: 3SV6). The expanded view illustrates the binding mode of telaprevir (green) within the active site, where hydrogen bonds are formed with His1057 and Gly1137 (distances shown in angstroms). In contrast, Ser1139 forms a covalent bond with the inhibitor, mimicking the tetrahedral intermediate of peptide bond hydrolysis. The structural zinc ion (orange sphere) maintains the architectural integrity of the catalytic pocket, ensuring the proper positioning of active site residues for substrate recognition and catalysis. (**C**) Structure of the NS3 helicase domain in complex with a single-stranded DNA (ssDNA, orange) and the non-hydrolyzable ATP analog ADP·AlF_4_^−^ (salmon) (PDB ID: 3KQL). The helicase domain contains several conserved sequence motifs (I–Y) that coordinate nucleic acid binding and ATP hydrolysis, essential for viral RNA unwinding during replication.

The NS3 protein is a key multifunctional protein in the HCV life cycle, consisting of an N-terminal serine protease domain and a C-terminal helicase domain. It forms a heterodimeric complex with the NS4A cofactor (NS3/4A) [12]. This NS3 protease (NS3P) belongs to the trypsin/chymotrypsin protease superfamily, and its proteolytic activity depends on the catalytic triad (His1057, Asp1081, Ser1139) and the oxyanion hole (formed by the backbone amides of residues such as Gly1137 and Ser1139) (Figure 2A,B) [29,30]. The binding of NS4A to NS3 significantly enhances its enzymatic activity. The central hydrophobic segment of NS4A inserts into the NS3 protease domain. This insertion forms a stabilizing β-strand augmentation. As a result, the catalytic fold is completed and rigidified. This structural stabilization maintains the catalytic triad in an optimal configuration for proteolysis [4]. Structurally, the NS3 protein adopts a characteristic double β-barrel fold: the N-terminal subdomain consists of an eight-stranded β-barrel and a conserved α-helix, while the C-terminal subdomain contains six β-strands [31] (Figure 2A). A zinc ion is located in the C-terminal subdomain and is essential for maintaining the structural stability of the protease and the relative spatial orientation of the adjacent two β-barrels. The zinc coordination site involves three conserved cysteine residues (Cys1097, Cys1099, and Cys1145) that form a partial tetrahedral geometry around the zinc ion, with cysteine sulfur-to-zinc distances of 2.0–2.5 Å. His1149 plays a context-dependent role in zinc coordination. In most cases, a water molecule occupies the fourth coordination site and lies within hydrogen-bonding distance of the His1149 side chain. Alternatively, His1149 can directly coordinate the zinc ion. In this configuration, the imidazole ring shields the zinc from solvent while remaining poised for coordination [14,32]. Point mutation studies have demonstrated that removal of any of these four residues (Cys1097, Cys1099, Cys1145, or His1149) negatively impacts NS3P processing activity, confirming their functional importance (Figure 2) [30,33,34].

### 3.2. Helicase Domain

The NS3 helicase domain belongs to superfamily 2 (SF2) helicases and exhibits a characteristic three-subdomain organization common to the DExH/D-box family members. The HCV NS3 protein utilizes energy derived from ATP hydrolysis to move along nucleic acids (RNA/DNA) in a 3′→5′ direction, unwinding double-stranded RNA or removing associated proteins. Structurally (Figure 2C), the helicase comprises three subdomains arranged around a central cleft that accommodates single-stranded nucleic acid. Key conserved motifs (motifs I-Y) coordinate ATP binding, hydrolysis, and coupling of chemical energy to mechanical strand separation [36].

Although the helicase domain can function independently, the presence of the NS4A cofactor and the NS3 protease domain markedly enhances RNA binding, NTPase activity, and RNA unwinding. These effects indicate strong functional interdependence between the protease and helicase domains [28,37]. Consistently, the catalytic behavior of the full-length NS3/4A complex differs from that of the isolated helicase domain, providing further evidence for tightly coupled interdomain regulation. NS3 helicase structures are largely derived from isolated constructs. While mechanistically informative, they do not capture the regulatory effects of the protease domain and NS4A present in the intact NS3/4A complex.

The interface between the NS3 protease and helicase domains includes both direct structural contacts and substrate-mediated interactions. This interface is proposed to dynamically couple these two domains and may function as a regulatory switch for enzymatic activity. Structural and biochemical studies further suggest that HCV NS3/4A can adopt an extended conformation. This state, similar to that observed in flavivirus homologs, may represent the catalytically active form of the complex [27].

### 3.3. Molecular Docking and Dynamics Studies

In terms of inhibitor binding, the active site region includes not only the catalytic triad but also key residues such as Arg1155 and Ala1156, forming the substrate-binding groove and playing a central role in drug recognition and inhibition [18]. For example, the NS3/4A–telaprevir complex structure (PDB ID: 3SV6, resolution: 1.40 Å) is commonly used as a reference, in which telaprevir forms hydrogen bonds with His1057 and Gly1137, and a covalent bond with Ser1139 (Figure 2B) [9]. Most NS3 protease structures, particularly inhibitor-bound complexes, represent experimentally stabilized states. Inhibitor binding and the use of short NS4A peptides constrain the protease into defined active-site geometries rather than the full conformational ensemble sampled during polyprotein processing. Molecular docking and dynamics simulations further characterize the binding of novel inhibitors, including Compounds **141** and **186**. These compounds form a stable hydrogen-bonding network with the catalytic triad and residues such as Gly1137. Consistent with these interactions, they exhibit higher binding affinity and greater conformational stability than the reference compound. This enhanced stability is quantitatively supported by molecular dynamics simulations. In the protein complex, compounds **141** and **186** display lower Root Mean Square Deviation (RMSD) values (0.36 nm and 0.34 nm, respectively) than the reference compound (0.47 nm). These reduced RMSD values indicate a more stable binding configuration [9].

Structural biology methods continue to advance the field. For example, microcrystal electron diffraction (MicroED) is used to resolve the polymorphic structures of paritaprevir. These analyses reveal that the β-polymorph fits more favorably within the genotype 1b NS3/4A binding site (PDB: 3KEE). This structural insight provides a basis for the rational optimization of acyl sulfonamide inhibitors [13]. Docking analyses reveal that one conformation fits well within the active-site pocket. Hydrophobic interactions and hydrogen bonds stabilize the binding. These results further highlight how distinct polymorphs and macrocycle conformations can be exploited to optimize inhibitor design. Despite these advances, a high-resolution crystal structure of genotype 3 NS3/4A is still lacking. This limitation hampers detailed analysis of mutation-specific effects [12]. Consequently, further structural studies remain essential.

### 3.4. Limitations of Current NS3/4A Structural Models

Although numerous high-resolution structures of the HCV NS3/4A complex have provided critical insights into catalytic architecture, interdomain organization, and inhibitor binding, several limitations should be considered when extrapolating mechanistic conclusions. Most available structures are derived from genotype 1, particularly genotype 1b, including full-length NS3/4A and protease–inhibitor complexes (e.g., PDB IDs: 1CU1, 3SV6, 3KEE). Whereas high-resolution structures of full-length NS3/4A from clinically important non–genotype 1 strains, especially genotype 3, remain unavailable despite their distinct resistance profiles [9,12,29,30,31,35]. In addition, many structures rely on truncated constructs or isolated domains, such as the protease domain bound to short NS4A peptides or independently crystallized helicase–nucleic acid complexes, with flexible regions including interdomain linkers, NS4A segments, and surface-exposed loops frequently unresolved due to conformational heterogeneity or crystal packing constraints [27,31,35,36]. Furthermore, inhibitor-bound structures typically capture stabilized conformational states selected by covalent engagement or lattice effects, providing invaluable information for defining catalytic geometry and drug–protein interactions. However, they may not fully represent the dynamic conformational ensemble underlying substrate processing and RNA unwinding [9,13,27,29,36]. Together, these considerations underscore the need to integrate static structural snapshots with biochemical, mutational, and molecular dynamics analyses, as well as to obtain full-length NS3/4A structures from diverse genotypes, to more comprehensively define the mechanistic and pharmacological landscape of this multifunctional viral enzyme [12,18,27,28].

Overall, the reported structural diversity of NS3/4A reflects both functionally relevant conformations and differences imposed by experimental conditions. Mechanistic interpretation, therefore, requires distinguishing intrinsic functional states from experimentally captured snapshots.

## 4. The Application in Drug Design: Inhibition of the NS3/4A Complex

### 4.1. Evolution and Efficacy of DAAs

The NS3/4A protease is a pivotal target for Hepatitis C therapy. The development of its inhibitors has evolved from first-generation linear α-ketoamides (e.g., telaprevir) to second-generation macrocyclic compounds, and finally to the current third-generation agents (e.g., paritaprevir, voxilaprevir, glecaprevir). This evolutionary trajectory reflects a major shift in inhibitor design. Early agents are slowly reversible covalent ketoamides approved in 2011 for use with interferon and ribavirin. In contrast, current inhibitors are reversible and span diverse structural classes, including acyclic compounds, P1–P3 macrocycles, P2–P4 macrocycles, and P1–P3/P2–P4 bismacrocyclic architectures [38]. These modern inhibitors incorporate optimized structural elements. Key features include precise macrocycle positioning and modifications of the P4 capping group. Together, these changes markedly enhance metabolic stability and activity against resistant strains [4,39]. A key advancement in third-generation inhibitors is the strategic incorporation of fluorine at the P2^+^ and P1 moieties. This modification enhances both antiviral potency and resistance profiles. These effects arise from fluorine’s strong electronegativity and relatively small atomic radius [40]. The most recent approvals include inhibitors with cross-genotype activity. These compounds show improved efficacy against key resistance-associated mutations at positions A1156, R1155, and D1168. As a result, they enable interferon/ribavirin-free combination therapies with cure rates approaching 100% and shorter treatment durations [38]. Clinically, combination regimens incorporating these advanced NS3/4A protease inhibitors, such as sofosbuvir/velpatasvir/voxilaprevir (SOF/VEL/VOX). This regimen demonstrates excellent pan-genotypic efficacy [41]. In the pivotal POLARIS-1 trial, this triple combination achieves a sustained virologic response rate of 96%. The efficacy is observed in patients who have previously failed NS5A inhibitor-containing regimens. In parallel, the POLARIS-4 reports a 98% response rate in patients with prior DAA exposure but without NS5A inhibitor experience [42]. Mechanistically, these drugs act through two distinct modes. Some function as reversible covalent inhibitors that form bonds with the catalytic residue S1139. Others act as competitive non-covalent inhibitors that mimic the substrate transition state [4].

### 4.2. Resistance Challenges and Genotype 3 Specificity

Despite these advances, the NS3/4A region remains prone to resistance-associated substitutions. Third-generation inhibitors are designed to fit the conserved substrate envelope better, thereby reducing susceptibility to common mutations [4]. However, specific mutations still confer significant resistance phenotypes:

Single-drug resistance: Associated with mutations such as V1062L, D1194E/T, and S1148A/G/R.

Dual resistance: Mediated by R1181K, A1182V, and T1080S.

Triple resistance: The D1194A mutation, for example, confers resistance to simeprevir, faldaprevir, and asunaprevir [17,43].

**Figure 3:**
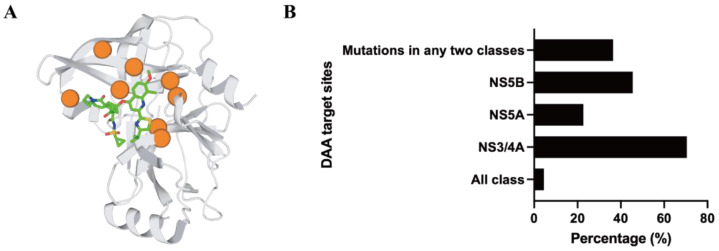
Structural context and prevalence of DAA resistance in HCV. (**A**) Resistance-associated substitutions mapped onto a representative NS3/4A protease–inhibitor complex (PDB: 3KEE) [29,45]. The protease is shown as a gray ribbon and the inhibitor as green sticks. Key resistance hotspots are highlighted as orange spheres to illustrate their spatial relationship to the inhibitor-binding pocket. Sulfur, yellow; nitrogen, blue; oxygen, red. (**B**) Prevalence of DAA resistance-associated substitutions across HCV genomic regions in Nepalese isolates [44]. 37 of 44 HCV genome sequences from Nepal harbored DAA-resistant mutations. NS3/4A region, 70.5%. NS5B, 45.5%. NS5A, 22.7%. Mutations in any two classes, 36.4%. All three regions, 4.5%.

Genotype 3 (GT3) remains particularly challenging, with lower response rates due to genotype-specific distribution patterns of mutations, such as V1055A and D1168Q [44]. To further illustrate the structural basis underlying the increased resistance of GT3, we compare key active-site residues of the NS3/4A protease between GT3 and the well-characterized GT1b (Table 2). Several amino acid differences are located within or adjacent to the inhibitor-binding pocket, providing a structural rationale for genotype-specific differences in protease inhibitor susceptibility. The magnitude of this challenge is highlighted in a recent study from Nepal, in which 70.5% of HCV sequences harbor DAA-associated substitutions in the NS3/4A region, a frequency substantially higher than that observed in the NS5A or NS5B regions (Figure 3) [44]. To place this enrichment in context, Figure 3 further integrates these prevalence data with a structural representation of the NS3/4A protease.

### 4.3. Emerging Strategies for Novel Inhibitor Discovery

To address resistance and the lack of specific therapies for GT3, recent research has integrated computational and experimental approaches to identify novel inhibitors (Table 3).

Structure-Based Virtual Screening: Utilizing homology modeling of GT3a NS3 and the ZINC database. Hussain et al. identify several candidate inhibitors, including N*-(2-(4-(piperidin-1-ylsulfonyl)benzylamino)ethyl)-*N*-(2,4,5-trichlorophenyl)methanesulfonamide* (TCP), N*-(2-(4-(morpholinosulfonyl)benzyl amino)ethyl)-*N*-(2,4,5-trichlorophenyl)methanesulfonamide* (TCM), and N*-(2,4-dichlorophenyl)-*N*-(2-(4-(piperidin-1-ylsulfonyl)benzylamino)ethyl)methanesulfonamide* (DCP). Experimental validation shows that TCP and DCP exhibit low cytotoxicity. In addition, surface plasmon resonance (SPR) measurements confirm that TCM binds to HCV-NS3 with high affinity (*K_D_* = 1.01 × 10^−7^ M) [18]. Other screening studies have also identified compounds, such as glucaric acid, with potential inhibitory activity [17].

Covalent and Polymorph Optimization: The novel covalent inhibitor cpd-217 (CHEMBL569970) maintains high affinity across 14 GT3 mutants by bonding with S1139, as confirmed by molecular dynamics simulations [12]. Additionally, structural studies using MicroED reveal that the β-polymorph of paritaprevir exhibits a superior binding affinity (−11.3 kcal/mol) compared with its α-polymorph [13].

Competitive and Allosteric Inhibition: The candidate molecule IJN19 is found to act as a competitive inhibitor, binding strongly to the D1168A mutant [1]. Beyond the active site, allosteric inhibitors (e.g., benzothiazole dimers) target the protease–helicase interface. When combined with macrocyclic inhibitors, these compounds show synergistic effects [4,46].

Dual-Function Inhibitors: Addressing the gap in helicase targeting, recent work has identified compounds that interact with the RNA-binding cleft (e.g., residues M1485, V1524, etc.). These dual-function candidates form stable hydrogen bonds and provide a strategy to simultaneously inhibit protease and helicase activities [47].

Natural Products and Ligand-Based Design: Natural compounds and their derivatives, such as EGCG and NP_024762, have been explored for their favorable safety profiles [14,48]. Furthermore, ligand-based tools, such as the HBCVTr (an end-to-end transformer with a deep neural network hybrid model for anti-HBV and HCV activity predictor from SMILES) method, facilitate rapid activity prediction from SMILES data [1].

PROTAC-Based Degradation: Unlike traditional DAAs that competitively inhibit the NS3/4A active site, PROTACs induce target degradation by recruiting E3 ubiquitin ligases to protein surface epitopes distant from the catalytic pocket. The first antiviral PROTAC, DGY-08-097, is developed by conjugating telaprevir (withdrawn due to resistance) to a cereblon (CRBN) E3 ligase ligand via a PEG linker [49]. This molecule recruits the CRL4CRBN complex to regions structurally independent of resistance-associated residues such as V1036, T1054, V1055, R1155, and A1156. Consequently, DGY-08-097 retains potent antiviral activity against telaprevir-resistant variants V1055A and A1156S—mutations that confer 6.9-fold and 25-fold resistance to telaprevir, respectively [49]. This spatial separation from the catalytic pocket imposes a higher genetic barrier: escape mutations must simultaneously abolish E3 ligase recruitment while preserving NS3/4A structural stability and enzymatic function, constraints far more stringent than simple mutations. Recent studies further demonstrate that macrocycle-based PROTACs targeting host dependency factors such as cyclophilin A also exhibit potent anti-HCV activity, expanding the PROTAC strategy beyond direct viral targets [4,50].

To provide a comprehensive overview of the diverse therapeutic strategies targeting NS3/4A, we have categorized current approaches by their mechanism of action and development status (Table 3). This classification illustrates the evolution from clinically approved DAAs to emerging modalities such as allosteric inhibition, PROTAC-mediated degradation, and helicase-specific inhibitors. The table highlights the mechanistic diversity available for next-generation anti-HCV drug development and provides a clear roadmap of the translational pipeline from preclinical discovery to clinical application.

**Table 3:** Categorization and Development Status of NS3/4A-Targeting Therapeutic Strategies.

Category	Representative Agents/Strategies	Mechanism of Action	Development Status	References
Approved DAAs	1st Gen: Telaprevir, Boceprevir	Linear α-ketoamides; reversible covalent inhibition by mimicking peptide substrate	Approved (First-generation)	[4,9,38]
	2nd Gen: Simeprevir, Asunaprevir	Macrocyclic compounds; competitive non-covalent inhibition with enhanced stability.	Approved (Second-generation)	[17,38,43]
	3rd Gen: Paritaprevir, Voxilaprevir, Glecaprevir	Pan-genotypic inhibitors with optimized P2+ and P1 fluorination to overcome resistance.	Approved (Standard of care)	[4,13,38,39,40]
Preclinical Inhibitors	TCP, TCM, DCP	Specifically optimized for GT3a; TCM shows high binding affinity (*K_D_* = 1.01 × 10^−7^ M).	Preclinical/Virtual screening	[9]
	Compounds **141**, **186**, Cpd-217	Novel covalent inhibitors maintaining high affinity across multiple GT3 mutants.	Preclinical/Computational	[18]
	Glucaric acid, EGCG, NP_024762	Natural products and derivatives with favorable safety profiles.	Preclinical screening	[14,17,48]
Allosteric Modulators	Benzothiazole dimers	Targets the protease-helicase interface to disrupt interdomain communication and allosteric coupling.	Research stage	[46]
PROTAC-based Strategies	DGY-08 series	Induces ubiquitination and proteasomal degradation of NS3/4A to bypass point-mutation-driven resistance.	Research stage/Emerging therapeutic modality	[4,49]
Helicase Inhibitors	RNA-binding cleft inhibitors (e.g., targeting M1485, V1524)	Disrupts ATP hydrolysis, nucleic acid binding, or mechanical unwinding; higher genetic barrier to resistance.	Research stage/Complementary strategy	[22,47,51,52,53,54]

## 5. The Application in Protein Engineering—Enhanced Activity of Helicases

### 5.1. Nanopore Sequencing Applications

Nanopore sequencing is a third-generation technology that enables real-time, single-molecule analysis with long read lengths and minimal sample preparation, and is widely used in both research and clinical settings [55]. Sequence information is derived from characteristic ionic current modulations as nucleic acids translocate through a nanopore.

A key technical limitation is the intrinsically rapid, sub-microsecond translocation of nucleic acids in the absence of regulation. This challenge has been effectively addressed by integrating ATP-dependent motor proteins—primarily polymerases and helicases—into nanopore platforms to control strand movement, a strategy that underlies current commercial sequencing systems.

Against this backdrop, the HCV NS3 helicase has emerged as a candidate motor protein owing to its high processivity, ATP-coupled translocation, and ability to unwind both RNA and DNA substrates. By coupling ATP hydrolysis to directional strand separation, NS3 can, in principle, act as an enzymatic brake to deliver single-stranded nucleic acids through the nanopore at kinetically accessible speeds.

Notably, targeted protein engineering has transitioned the NS3 helicase from a theoretical candidate to an experimentally validated tool in nanopore sequencing. Recent industrial disclosures, such as Patent CN 120230736 A (Beijing Qitan Technology Co., Ltd., Beijing, China) [56], provide empirical evidence that site-specific mutations can effectively fine-tune the enzyme’s binding affinity and kinetic stability. These engineered variants—moving beyond mere biochemical inference—demonstrate precise control over molecular translocation speeds, a prerequisite for high-resolution signal characterization on nanopore platforms.

Although most established nanopore platforms rely on polymerases or bacterial helicases such as Dda, Hel308 or UvrD, the NS3 helicase has been examined in biochemical studies and nanopore platforms as an alternative motor whose kinetic properties are amenable to protein engineering [57,58,59]. These efforts aim to expand the range of motor proteins available for nanopore sequencing and related single-molecule applications.

### 5.2. Biochemical Requirements of Helicase Activity

Regarding biochemical requirements, the NS3/4A complex exhibits a preference for Mn^2+^ for its helicase activity. MnCl_2_ is more effective than MgCl_2_ at all tested concentrations, with an optimal MnCl_2_ concentration ranging from 1.5 to 3 mM [60]. The optimal pH range for helicase activity is narrowly defined between 6.0 and 6.5. The NS3/4A complex can act on both RNA/DNA hybrid duplex substrates and DNA templates, except for M13 DNA/DNA substrates. The hydrolysis of high-energy phosphate bonds is the essential energy source for this activity; while NTPs and dNTPs can be utilized by NS3/4A, ATP provides the highest efficiency [60].

### 5.3. Engineering Strategies for Optimizing Helicase Activity

Interface mutations can modulate helicase activity and substrate binding. Certain mutants, exemplified by D1079A, Q1526A, and H1528A, display significantly enhanced ssDNA binding affinity. While other mutants, such as the triple helicase mutant (S1483A, M1485 and V1524A), exhibit markedly diminished binding strength but enhanced unwinding rates [27]. Beyond interface residues, site-specific mutations in the catalytic core and nucleic acid-binding cleft are instrumental in modulating translocation kinetics. For instance, mutations in the Walker A motif (e.g., T1212) can slow down ATP hydrolysis, thereby reducing the stepping frequency to improve signal resolution. Additionally, the aromatic residue W1501, which acts as a ‘molecular ratchet’ through base-stacking, can be mutated to fine-tune the motor’s grip on the substrate [36,61]. Taken together, these findings provide a foundation for rational engineering of NS3 helicase variants with optimized properties for biotechnological applications, including enhanced performance in nanopore sequencing assays where controlled unwinding rates and processivity are critical parameters. To further highlight the potential of NS3 in biotechnological applications, a comparison with the commonly used Phi29 DNA polymerase is summarized in Table 4.

## 6. Discussion

The protease domain acts as a ‘kinetic brake,’ optimizing the trade-off between helicase speed and processivity [27]. This regulation synchronizes unwinding with NS5B-mediated RNA synthesis, which is critical for maintaining replication fidelity. Mechanistically, a controlled unwinding pace prevents the premature disruption of essential RNA secondary structures, such as the IRES and 3′ UTR, ensuring stable translation and replication initiation [61]. These biological constraints explain why interface-disrupting mutants—despite their increased catalytic speed—exhibit significantly reduced viral fitness in replicon models [62]. This implicates the full-length protein in constraining its activity through a physiologically relevant kinetic brake.

Evidence for this regulatory mechanism comes from multiple experimental systems. In vitro biochemical assays comparing isolated helicase domains with full-length NS3 constructs first established this paradigm [51,61]. Structural analyses subsequently reveal conformational coupling between the protease and helicase domains [31,36,63]. Critically, cell-based replication studies validate its functional relevance. Domain-deletion mutants and interface-disrupting substitutions alter replication kinetics and reduce viral fitness in cultured hepatocytes [62,64].

This convergence of evidence underscores a broader principle in viral enzymology. Enzymatic efficiency is governed not only by the active-site pocket but also by distal structural communication. This offers attractive leverage points for mechanism-based antiviral design.

Concurrently, the advent of DAAs has ushered in a transformative shift in HCV therapy, achieving high sustained virologic response (SVR) rates with acceptable safety. However, the clinical utility is constrained by resistance-associated substitutions (RASs), particularly within the NS3/4A protease domain, which can erode therapeutic efficacy and lead to treatment failure. Genotype-specific RASs—especially those affecting genotypes 1 and 5—highlight the need for routine pre-treatment resistance profiling and dynamic monitoring during therapy [43].

Although combination regimens mitigate resistance risk, they also raise concerns regarding pharmacokinetic drug–drug interactions that warrant systematic clinical oversight [6].

In contrast to protease inhibitors, resistance evolution against helicase-targeting strategies is expected to be intrinsically constrained. The NS3 helicase is subject to strong functional coupling between ATP hydrolysis, nucleic acid binding, and interdomain communication, leaving limited mutational space for resistance without severe fitness costs [22,51,52]. Accordingly, inhibitors that disrupt helicase activity indirectly—by perturbing conformational dynamics or protease–helicase coupling—are predicted to impose a higher genetic barrier to resistance than active-site-directed protease inhibitors.

High-throughput screens targeting NS3 helicase function, therefore, represent a complementary strategy to existing DAAs, with the dual potential to bypass protease inhibitor resistance and to exploit evolutionarily constrained regulatory mechanisms [53,54].

## 7. Conclusions and Perspectives

### 7.1. Conclusions

The NS3/4A protease serves as a critical target in HCV therapy, and the development of its inhibitors has achieved remarkable success in clinical applications. However, drug development continues to face significant challenges due to the increasing prominence of resistance in genotype 3 (GT3) and the ongoing emergence of mutant strains. Although most approved NS3/4A inhibitors are designed to be broadly active across HCV genotypes, their reduced efficacy against GT3 highlights the need for genotype-specific optimization.

Currently, by integrating multi-pronged strategies—including structural biology, virtual screening, allosteric inhibition, and natural product mining—researchers have made serial progress in developing protease inhibitors specifically targeting GT3-associated structural and sequence features. For instance, high-throughput screening approaches combining computational simulations with experimental validation have identified several candidate molecules, such as TCP, TCM, and DCP, which exhibit favorable binding affinity, pharmacokinetic properties, and safety profiles, supporting their potential as GT3-focused inhibitors rather than universally pan-genotypic agents [18,65].

Although existing direct-acting antiviral drugs demonstrate significant overall efficacy in HCV treatment, resistance remains a major obstacle in clinical management. Future research should therefore balance genotype-specific inhibitor design with broadly applicable strategies that target conserved elements of NS3/4A by integrating high-resolution structural information with computer-aided drug design and advancing emerging approaches such as allosteric inhibition, natural product screening, and PROTACs. In conclusion, combining genotype-tailored and pan-genotypic strategies offers a rational path toward developing next-generation anti-HCV agents capable of overcoming genotype-dependent resistance.

### 7.2. Perspectives

**Figure 4:**
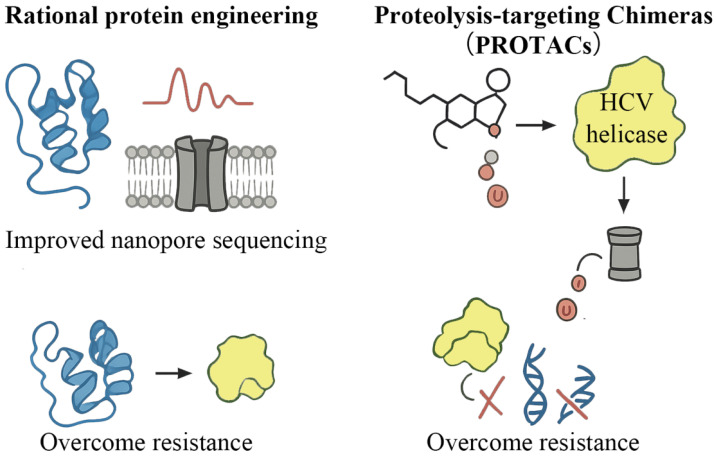
Future research landscape for the HCV NS3/4A proteins: Rational protein engineering and Proteolysis-Targeting Chimeras (PROTACs). This schematic diagram illustrates two powerful, next-generation molecular strategies—Rational Protein Engineering and PROTACs—for tackling the critical viral factor, the HCV NS3/4A proteins, outlining a roadmap for future therapeutic and biotechnological advancements, particularly focused on addressing drug resistance.

Rational protein engineering, increasingly guided by artificial intelligence (AI)-driven structural modeling, has been leveraged to improve nanopore sequencing performance by enhancing pore–analyte interactions and signal resolution (Figure 4). In the quest for next-generation anti-HCV therapeutics, PROTAC represents a mechanistically distinct modality. By inducing ubiquitination and subsequent proteasomal degradation of viral or host cofactor proteins, PROTAC offers a strategy to bypass resistance mechanisms typically driven by point mutations that compromise the efficacy of classical small-molecule inhibitors. DGY-08-097 exemplifies this advantage: it retains potent antiviral activity against V1055A and A1156S variants that confer 6.9- and 25-fold resistance to telaprevir, respectively [49]. By recruiting E3 ligases to surface epitopes rather than competing for active-site binding, PROTACs circumvent mutations at V1036, T1054, V1055, R1155, and A1156—residues that directly contact conventional inhibitors. This spatial separation imposes a higher genetic barrier: resistance requires simultaneous disruption of ligase recruitment and preservation of catalytic function (Figure 4) [4,49].

Parallel efforts have explored non-degradative strategies, including miRNA modulation, immunotherapy, and vaccine development; however, extensive HCV genetic heterogeneity continues to limit vaccine efficacy, leaving direct-acting antivirals as the clinical mainstay [4].

Looking forward, three priority challenges must be addressed. First, the in vivo specificity, safety, and durability of PROTAC-based antiviral strategies remain insufficiently characterized. Second, it is still unclear which conserved structural features of NS3/4A can be optimally targeted to simultaneously maximize potency and genetic barriers to resistance. Third, the practical integration of PROTAC and allosteric modalities into scalable high-throughput screening frameworks has yet to be established. Resolving these issues will be critical for translating next-generation, resistance-resilient anti-HCV strategies into clinical reality.

## Figures and Tables

**Table 1:** Summary of HCV NS3/4A Functional Regulation and Inter-Domain Communication [27]. This table summarizes key experimental findings concerning the regulation of HCV NS3/4A helicase/ATPase activities, highlighting the roles of the inter-domain interface and the NS3 protease domain in modulating helicase function.

Activity Category	KEY Observation	Regulatory Mechanism
**D** **NA unwinding activity**	Disrupting the structural interface will significantly enhance the DNA-unwinding rate.	The activity of the interface mutant (e.g., Triple Hel, S1483A-M1485A-V1524A mutation) is up to 11.6-fold higher than that of the wild-type full-length protein. Isolated NS3 helicase unwinds faster than the full-length NS3/4A (up to 5.7-fold).
	The processivity is consequently affected.	The protease domain enhances the helicase’s processivity under single-turn unwinding conditions. Conversely, mutations that disrupt the direct interface increase overall activity but compromise processivity.
**R** **NA unwinding activity**	The RNA unwinding activity of the full-length protein is significantly higher than that of the isolated helicase domain. Specifically, the isolated NS3 helicase unwinds RNA approximately 10-fold more slowly than the full-length scNS3/4A.	The enhanced rate is attributed to the presence of the protease domain rather than mediation through the interdomain interface.
	Mutations have negligible effects at the interface.	The protease domain may function as a helicase cofactor, stabilizing protein-RNA interactions and modulating RNA unwinding activity and persistence. There is a potential direct interaction between RNA and the protease domain.
**ssDNA Binding Affinity**	Interface mutations modulate ssDNA affinity.	Notably, certain mutants, exemplified by D1079A, Q1526A, and H1528A, display significantly enhanced binding affinity. In contrast, other mutants, such as Triple Hel, exhibit markedly diminished binding strength.
**RNA-stimulated ATPase activity**	Not affected by interface mutation.	This result is similar to the results of the RNA unwinding experiment.

**Table 2:** Comparison of key amino acid residues in the NS3/4A protease binding pocket between Genotypes 1b and 3a.

Residue Position	Genotype 1b	Genotype 3a	Structural and Functional Impact on Inhibitor Binding
1036	Val (V)	Leu (L)	Increases the hydrophobicity of the S1 pocket, potentially affecting the fit of early-generation protease inhibitors (PIs).
1123	Arg (R)	Thr (T)	Significant shift: Replaces a long, positively charged side chain with a shorter, polar one, altering the hydrogen-bonding network in the S4 pocket.
1132	Ile (I)	Leu (L)	Subtle rearrangement of the hydrophobic environment within the S2 pocket, affecting the orientation of the P2 moiety.
1158	Val (V)	Ile (I)	Positioned near the S3/S4 boundary; the bulkier side chain in GT3a may influence the binding of macrocyclic inhibitors.
1168	Asp (D)	Gln (Q)	Critical distinction: GT3a naturally carries Gln1168, which lacks the negative charge of Asp1168. This eliminates key electrostatic interactions (salt bridges) with P2 substituents, drastically reducing the potency of macrocyclic inhibitors like BILN 2061.
1175	Met (M)	Leu (L)	Located near the active site; contributes to the overall stability and minor conformational shifts between the two genotypes.

Note: While key binding pocket residues vary, the catalytic triad (H1057, D1081, S1139) remains highly conserved across all genotypes to ensure basic proteolytic efficiency.

**Table 4:** Comparison between engineered NS3 and Phi29 DNA polymerase for nanopore applications.

Feature	Phi29 DNA Polymerase	HCV NS3 Helicase
Substrate Compatibility	Primarily DNA	Both DNA and RNA [61].
Driving Force	DNA Synthesis	ATP-dependent Unwinding
Primer Requirement	Requires a primer	Primer-independent
Key Advantage	Extreme processivity for DNA	Superior for direct RNA sequencing [62]

## Data Availability

No new data were created or analyzed in this study. Data sharing is not applicable to this article.

## Abbreviations

The following abbreviations are used in this manuscript:

ADRP	Adipose differentiation-related protein
CCTα	CTP-phosphocholine-cytidyl transferase alpha
COVID-19	Coronavirus disease 2019
DAAs	Direct-acting antiviral agents
DCP	N-(2,4-dichlorophenyl)-N-(2-(4-(piperidin-1-ylsulfonyl)benzylamino)ethyl)methanesulfonamide
FPN1	Ferroportin 1
GT3	Genotype 3
HBCVTr	An end-to-end transformer with a deep neural network hybrid model for anti-HBV and HCV activity predictor from SMILES
HCV	Hepatitis C virus
MAVS	Mitochondrial antiviral-signaling protein
MicroED	Microcrystal electron diffraction
NS3P	NS3 protease
PIs	Protease inhibitors
PROTACs	Proteolysis-targeting chimeras
RASs	Resistance-associated substitutions
RIG-I/MAD5	Retinoic acid-inducible gene I/melanoma differentiation-associated protein 5
RMSD	Root Mean Square Deviation
SF2	Superfamily 2
SPG20	Spartin
SPR	Surface plasmon resonance
SVR	Sustained virologic response
TCM	N-(2-(4-(morpholinosulfonyl)benzyl amino)ethyl)-N-(2,4,5-trichlorophenyl)methanesulfonamide
TCP	N-(2-(4-(piperidin-1-ylsulfonyl)benzylamino)ethyl)-N-(2,4,5-trichlorophenyl)methanesulfonamide
TRIF	TIR-domain-containing adapter-inducing interferon-β
